# Clinical Factors Associated with Long-Term Complete Remission versus Poor Response to Chemotherapy in HIV-Infected Children and Adolescents with Kaposi Sarcoma Receiving Bleomycin and Vincristine: A Retrospective Observational Study

**DOI:** 10.1371/journal.pone.0153335

**Published:** 2016-04-15

**Authors:** Nader Kim El-Mallawany, William Kamiyango, Jeremy S. Slone, Jimmy Villiera, Carrie L. Kovarik, Carrie M. Cox, Dirk P. Dittmer, Saeed Ahmed, Gordon E. Schutze, Michael E. Scheurer, Peter N. Kazembe, Parth S. Mehta

**Affiliations:** 1 Department of Pediatrics, Division of Hematology, Oncology, and Hematopoietic Stem Cell Transplantation, New York Medical College, Valhalla, New York, United States of America; 2 Department of Pediatrics, Section of Hematology and Oncology, Baylor College of Medicine, Houston, Texas, United States of America; 3 Baylor College of Medicine Children’s Foundation Malawi, Lilongwe, Malawi; 4 Texas Children’s Cancer and Hematology Centers, Houston, Texas, United States of America; 5 Department of Dermatology, University of Pennsylvania, Philadelphia, Pennsylvania, United States of America; 6 Department of Pediatrics, Loyola University Chicago, Stritch School of Medicine, Maywood, Illinois, United States of America; 7 Department of Microbiology and Immunology, Lineberger Comprehensive Cancer Center, University of North Carolina, Chapel Hill, North Carolina, United States of America; 8 Department of Pediatrics, Baylor College of Medicine, Houston, Texas, United States of America; University of Southern California Keck School of Medicine, UNITED STATES

## Abstract

Kaposi sarcoma (KS) is the most common HIV-associated malignancy in children and adolescents in Africa. Pediatric KS is distinct from adult disease. We evaluated the clinical characteristics associated with long-term outcomes. We performed a retrospective observational analysis of 70 HIV-infected children and adolescents with KS less than 18 years of age diagnosed between 8/2010 and 6/2013 in Lilongwe, Malawi. Local first-line treatment included bleomycin and vincristine plus nevirapine-based highly active anti-retroviral therapy (HAART). Median age was 8.6 years (range 1.7–17.9); there were 35 females (50%). Most common sites of presentation were: lymph node (74%), skin (59%), subcutaneous nodules (33%), oral (27%), woody edema (24%), and visceral (16%). Eighteen (26%) presented with lymphadenopathy only. Severe CD4 suppression occurred in 28%. At time of KS diagnosis, 49% were already on HAART. Overall, 28% presented with a platelet count < 100 x 10^9^/L and 37% with hemoglobin < 8 g/dL. The 2-year event-free (EFS) and overall survival (OS) were 46% and 58% respectively (median follow-up 29 months, range 15–50). Multivariable analysis of risk of death and failure to achieve EFS demonstrated that visceral disease (odds ratios [OR] 19.08 and 11.61, 95% CI 2.22–163.90 and 1.60–83.95 respectively) and presenting with more than 20 skin/oral lesions (OR 9.57 and 22.90, 95% CI 1.01–90.99 and 1.00–524.13 respectively) were independent risk factors for both. Woody edema was associated with failure to achieve EFS (OR 7.80, 95% CI 1.84–33.08) but not death. Univariable analysis revealed that lymph node involvement was favorable for EFS (OR 0.28, 95% CI 0.08–0.99), while T_1_ TIS staging criteria, presence of cytopenias, and severe immune suppression were not associated with increased mortality. Long-term complete remission is achievable in pediatric KS, however outcomes vary according to clinical presentation. Based on clinical heterogeneity, treatment according to risk-stratification is necessary to improve overall outcomes.

## Introduction

Kaposi sarcoma (KS) is the most common human immunodeficiency virus (HIV)-associated malignancy in children and adolescents in sub-Saharan Africa.[1] KS is caused by human herpesvirus-8 (HHV-8) infection, a virus with prevalence rates that vary geographically.[2] Eastern and central Africa in particular have the highest prevalence of HHV-8 infection in the world.[3–9] Consequently, KS has become an important and widespread complication of the HIV epidemic in sub-Saharan Africa, affecting not only adults, but children and adolescents as well. While clinical descriptions and treatment paradigms for adult KS have been well established, there exist only a few clinical descriptions of pediatric KS cohorts in Africa.[10–14] Standardized therapeutic strategies and risk factors associated with survival outcomes for children have yet to be established. Furthermore, since the largest published series on HIV-associated malignancies from the United States or Europe reported only eight pediatric KS patients from 1978–1996 in the AIDS-Cancer Match Registry Study Group, the pre-highly active anti-retroviral therapy (HAART) era in the western world fails to serve as a guide.[15–20]

HHV-8, or Kaposi sarcoma associated herpesvirus (KSHV), is endemic in eastern and central Africa with reported prevalence rates in children of 25–60% in Malawi, Uganda, and Tanzania; the HHV-8 seroprevalence in adults ranges between 60–90% in the same region.[3–5, 8, 21–24] HHV-8 transmission often occurs during childhood from mother to child or between siblings through exposure to oral secretions in endemic regions of the world.[9, 25–27] Therefore, with the onset of HHV-8 infection occurring at any point during childhood, KS can develop at any time in the life of an HIV-infected child—either as a consequence of primary HHV-8 infection or a secondary viral re-activation.

Pediatric KS is distinct from adult disease in many ways. Some of the unique features of pediatric KS in sub-Saharan Africa include the high rates of lymph node involvement, inadequate response to HAART alone, and lack of prognostic significance in the AIDS Clinical Trial Group (ACTG) TIS staging classification.[10, 11, 13, 28, 29] One-year overall survival (OS) for most pediatric cohorts is approximately 40%, and risk factors predicting failure to respond to chemotherapy plus HAART have yet to be determined.[10–14, 29]

Despite high mortality rates in pediatric KS patients, long-term (i.e. > 3 years) complete remission (CR) has been achieved in HIV-infected children with KS in Malawi through the combination of chemotherapy and long-term HAART.[10] We evaluated the clinical characteristics of HIV-infected children and adolescents with KS in Lilongwe, Malawi and identified specific clinical factors that influence survival outcomes in patients receiving bleomycin and vincristine (BV) in combination with HAART.

## Methods

### Study Setting

We retrospectively analyzed the medical records of 70 consecutive HIV-infected children and adolescents with KS between August 2010 and June 2013 at the Baylor College of Medicine Children’s Foundation Malawi Clinical Centre of Excellence (MW-COE) on the campus of Kamuzu Central Hospital (KCH) in Lilongwe, Malawi, a tertiary care facility that serves a catchment area of approximately 5–8 million people. The primary clinical team included one pediatric hematologist-oncologist, two specialized clinical officers, two staff nurses and additional support staff comprised of social workers, pharmacy technicians, and clinic volunteers. Data were extracted from an electronic medical record system and standardized treatment flow sheets; patient health information was protected for confidentiality.

### Diagnostic and Therapeutic Strategies

Standard clinical evaluation included a physical exam, full blood count, CD4 count analysis, and chest x-ray (CXR). Standardized KS diagnosis/treatment forms were used to maintain consistency in evaluation and documentation. Due to resource constraints, there were occasions when standard diagnostic studies were not available. There was very limited availability of HIV viral load analysis throughout the study period.

Biopsies were only performed in patients where a clinical diagnosis was uncertain as the availability of pathology resources was limited until 2013. Biopsy specimens were initially processed for histological evaluation with hematoxylin and eosin (H&E) stains at the University of Pennsylvania in Philadelphia, USA. Starting in 2013, through support from the University of North Carolina Project–Malawi, specimens were processed and evaluated at the local KCH pathology laboratory.[30] Morphology of the malignant cells was determined on H&E and confirmed with the HHV-8 latency-associated nuclear antigen (LANA) immunohistochemical stain.

The up-front chemotherapy regimen included bleomycin 15 units/m^2^ via intramuscular (IM) or intravenous (IV) injection and vincristine 1.4 mg/m^2^ via IV injection. For naïve patients, HAART was initiated within 2 weeks of chemotherapy. This standard practice is based on extrapolation from the standard approach provided by the Malawi National HAART Guidelines to initiate anti-tuberculosis treatment within 2 weeks of HAART initiation in patients with newly diagnosed HIV and tuberculosis co-infection.[31] BV was given for a total of 8 cycles in two phases: induction phase (4 cycles given every 2 weeks), followed by a consolidation phase (4 additional cycles given every 4 weeks). In 2012 doxorubicin became available, and the addition of doxorubicin to BV (ABV) served as a salvage regimen (doxorubicin 35 mg/m^2^ IV, bleomycin 10 units/m^2^ IM, and vincristine 1.4 mg/m^2^ IV, administered every 3 weeks). Cumulative dosing of bleomycin and doxorubicin was tallied for each patient; the total lifetime limit of bleomycin was established as 250 units/m^2^ and for doxorubicin as 300 mg/m^2^. The third line chemotherapeutic option was paclitaxel 75 mg/m^2^ IV infusion every 3 weeks based upon a modified protocol from our experience in Botswana.[32]

General guidelines for chemotherapy included delaying and/or dose-adjusting chemotherapy for absolute neutrophil count (ANC) < 1,000 cells/mm^3^ and/or platelet count < 100 x 10^9^/L. However, because a significant percentage of patients presented with cytopenias as part of the original clinical picture of their KS, the initial two cycles of chemotherapy were given at full-dose regardless of blood counts. For patients experiencing multiple occurrences of chemotherapy-induced cytopenias, adjustments were made to the co-trimoxazole regimen and/or patients were switched off of zidovudine (AZT) containing-HAART.

HAART was based on national guidelines in Malawi with nevirapine (NVP)-based first line regimens. Prior to 2011, first-line HAART consisted of stavudine (d4T), lamivudine (3TC), and NVP. Thereafter, AZT replaced d4T. Protease inhibitor-based HAART was the standard second-line option with lopinavir-boosted ritonavir combined with 3TC and either abacavir or tenofovir during the study period. Patients receiving anti-tuberculosis treatment for co-infection were treated with efavirenz-based regimens instead of nevirapine. Additional modifications of the standard HAART regimen were available for patients with specific contra-indications and/or clinical scenarios. Daily co-trimoxazole was given for prophylaxis against pneumocystis jiroveci according to World Health Organization (WHO) guidelines. Financial support for transportation was provided to patients requiring financial assistance.

Resources for supportive care were limited but consistently available. Moderate-severe malnutrition was treated according to WHO guidelines with peanut butter-based supplements and/or milk-based formulas. Anti-bacterial medicines included parenteral ceftriaxone and the following oral antibiotics: ciprofloxacin, amoxicillin, metronidazole, azithromycin, and co-trimoxazole. Tuberculosis was treated according to WHO guidelines utilizing a 4-drug RHZE (rifampicin, isoniazid, pyrazinamide, and ethambutol) initial phase, followed by a 2-drug RH (rifampicin and isoniazid) continuation phase. Streptomycin was available for patients requiring re-treatment. The only anti-fungal medicine available was oral fluconazole; there were no medicines available for the treatment of invasive mold infections. There were no anti-viral agents at our disposal other than HAART. Anti-parasitic agents included artesunate-based oral medicines and parenteral quinine for malaria, albendazole and/or mebendazole (and metronidazole) for intestinal parasite infections, and praziquantel for schistosomiasis. Oral co-trimoxazole was given for treatment of pneumocystis jiroveci pneumonia as well as toxoplasma infections. Cultures of body fluids and advanced diagnostic methods of isolating infectious organisms were not available, therefore opportunistic infections were clinically diagnosed and treated based upon WHO clinical guidelines. We generally maintained a low threshold to empirically treat opportunistic infections with the available supportive care medicines. Whole blood transfusion was usually available from the Malawi Blood Bank, however platelets for transfusion were extremely difficult to obtain.

### Clinical Definitions

The ACTG staging criteria were applied to all patients classifying tumor extent (T), immune status (I) and systemic illness (S) based upon the guidelines established for adults with KS.[33] However, because in our experience the ACTG staging criteria did not consistently provide prognostic relevance for pediatric patients, we also carefully documented the extent of KS involvement in each patient using standardized data collection forms.[10]

We differentiated sites of KS involvement based upon the following definitions:

- Skin: hyperpigmented macules/patches or papules
- Oral: hyperpigmented macules/patches or papular/nodular lesions of the oral mucosa
- Woody Edema: firm, non-pitting edema that typically occurs in the extremities and/or pubic region, its texture resembling that of tree bark
- Lymphadenopathy: bulging, firm, non-tender, enlarged lymph nodes measuring > 2 cm in diameter, and not attributable to another cause of lymphadenopathy
- Subcutaneous Nodules: non-hyperpigmented nodules that are distinctly subcutaneous and may occur as isolated nodules or as clusters of knotty lesions in the subcutaneous tissue, the latter often associated with underlying woody edema
- Facial Edema: non-woody, soft edema of the face, often in the peri-orbital region
- Other: includes exophytic masses and conjunctival lesions

In the absence of bronchoscopy and endoscopy, our definitions of pulmonary and abdominal cavity visceral KS were established considering the limitations in resources.

- Pulmonary: in those patients with KS and a CXR demonstrating reticulo-nodular infiltrates and/or large pleural effusions not attributable to other pulmonary pathology (including demonstration of a failure to improve on empiric anti-tuberculosis and other anti-bacterial treatment). Serosanguineous effusions without evidence of infectious etiology were considered confirmatory for pulmonary KS. Additionally, a clinical diagnosis of upper airway pulmonary KS is defined in patients with KS and clinical signs of upper airway obstruction that improve with chemotherapy.
- Abdominal Cavity: in those patients with KS and persistent bloody stools not attributable to other causes of hemorrhagic colitis (ie demonstration of failure to improve on empiric anti-bacterial and anti-parasitic treatment). Also, in patients with KS and severe ascites not attributable to another disease process.

Definitions for disease response to chemotherapy were based upon previously established criteria from the ACTG.[34] The ACTG criteria define CR as the absence of any detectable residual lesions. CR was always clinically determined, as patients did not have confirmatory biopsies performed. However, CR was uniformly validated by the lack of progression in this cohort with consistent long-term follow-up. Relapse was defined as the recurrence of KS after having previously achieved CR. We have defined an additional response group called very good partial response (VGPR), in which patients achieve a subjective measure of at least 90% reduction in the size of all lesions. Induction failure was defined as failing to achieve a VGPR or CR after induction phase BV. For the survival analyses, event was defined as the occurrence of relapse, disease progression, failure to achieve CR, or death from any cause.

Immunological definitions from WHO guidelines were used, establishing severe and advanced immunosuppression as follows for the following age groups: infants younger than 12 months, a CD4% of < 20% and < 25% respectively. For children aged 1–5 years, a CD4% of < 15% and < 20% respectively, and for children 5 years and older, an absolute CD4 count < 200 and < 350 respectively. Immune reconstitution inflammatory syndrome (IRIS) was defined as occurrence of new-onset or worsening KS within 6 months of the initiation of HAART in patients who have not received chemotherapy. Virologic suppression of HIV is an important criteria to monitor in all patients, however due to limitations in resources, information on HIV viral load was only available in a few select clinical scenarios over the time period of the study, and therefore not discussed. The diagnosis of tuberculosis was clinical and WHO definitions of severe acute malnutrition were used.[35]

### Statistical Analysis and Ethical Considerations

Demographic, disease and treatment information were described by standard descriptive statistics. Dichotomous variables were analyzed using the χ^2^ test, and continuous variables were analyzed using the t-test for two outcome categories and reported as mean and standard deviation. Univariate and multivariable analyses were done to evaluate the risk of death and the risk of failure to achieve event-free survival (EFS). Analyses were conducted utilizing either standard logistic regression or penalized maximum likelihood regression if there were any variables for which a subset of analyzed subjects were not exposed, i.e., zero cells in a contingency table.[36] The stepwise backward selection procedure (p < 0.05) was used to build the most parsimonious multivariable model for each outcome.

Both OS and EFS are reported using standard Kaplan-Meier curves. All statistical analyses were done using STATA^TM^, version 14 (StataCorp LP, College Station, TX, USA). Ethics committee approval was obtained for this retrospective review from both the Malawi National Health Science Research Committee as well as the Baylor College of Medicine Institutional Review Board for Human Subjects Research. Due to the retrospective nature of this study, informed consent was not obtained for the review of medical records. However, data was anonymized and de-identified prior to analysis to maintain strict protection of confidentiality.

## Results

### Patient Characteristics

**Fig 1:**
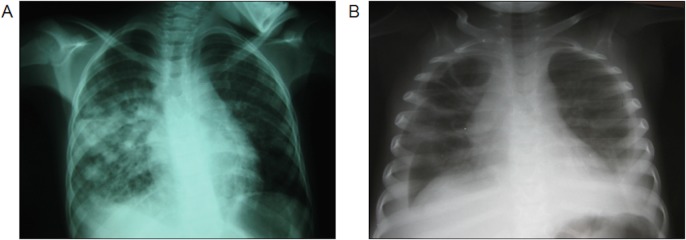
Representations of pulmonary KS on chest x-ray in patients with classic hyperpigmented KS skin lesions. (A) Multiple, scattered reticulo-nodular infiltrates in the right lung and (B) large pleural effusions.

**Fig 2:**
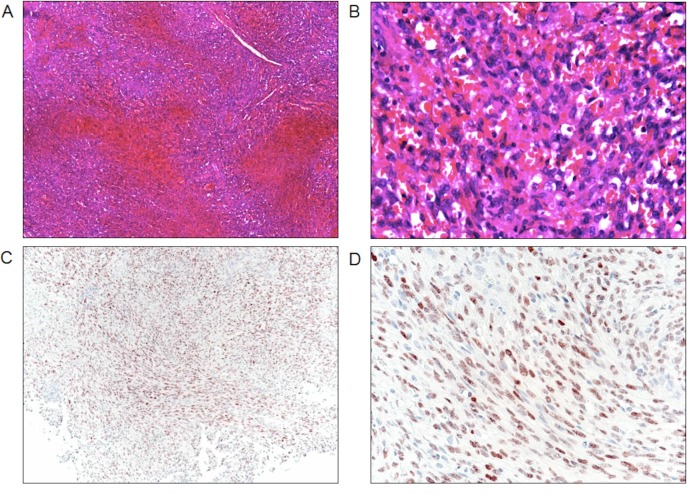
Morphology of KS in a lymph node biopsy. Complete effacement of the nodal architecture by a spindle cell infiltrate at (A) low and (B) high power on hematoxylin and eosin stain that are diffusely positive for the HHV-8 latency-associated nuclear antigen immunohistochemical stain shown at (C) low and (D) high power.

During the study period, 70 HIV-infected (presumed vertical transmission) children and adolescents < 18 years of age were diagnosed with KS at the MW-COE and KCH. (Table 1) Using a denominator of children initiated on HAART at the MW-COE as a proxy for number of new pediatric HIV diagnoses during that time period, KS represented 5.2% (70/1,359) of children and adolescents newly diagnosed with HIV infection. There were 35 females (50%) and the median age at the time of KS diagnosis was 8.6 years (range 1.7–17.9). The most common sites of KS clinical involvement were lymph node (74.3%) and skin (58.6%). Visceral disease occurred in 16%. Notably, 18 patients (25.7%) presented with *lymphadenopathy only*, and no classic KS lesions of the skin, oral mucosa, or woody edema. Fig 1 demonstrates the radiographic presentations of pulmonary KS in our patients. Seven patients (10%) presented with > 20 hyperpigmented skin/oral lesions in a widespread, “disseminated” distribution all over the body. The inguinal region, including the groin/inner thigh, was involved in 58 patients (82.9%), either in the form of lymphadenopathy, skin lesions, subcutaneous nodules, or woody edema. Diagnosis was pathologically confirmed in 14 patients (20%); Fig 2 demonstrates a representation of the histologic and immunohistochemical lymph node biopsy findings. Baseline CXR was obtained at the time of KS diagnosis in 51 of 70 patients (72.9%).

**Table 1:** Clinical Characteristics of HIV-infected Children and Adolescents with Kaposi Sarcoma.

Number of Patients		70
Gender		Female 35 (50%)
Median Age (range)		8.6 years (1.7–17.9)
Pathology Confirmation		14 (20%)
Clinical Site of KS Involvement		
	Lymph Node	52 (74.3%)
	Hyperpigmented Skin Lesions	41 (58.6%)
	Non-Hyperpigmented Subcutaneous Nodules	23 (32.9%)
	Oral	19 (27.1%)
	Woody Edema	17 (24.3%)
	Facial Edema	11 (15.7%)
	Pulmonary	8 (11.4%)
	Abdominal Visceral	3 (4.3%)
	Lymph Node ONLY, biopsy-proven in 13 of 18 (72.2%)	18 (25.7%)
	Inguinal Region Involvement (any type of lesion)	58 (82.9%)
	More than 20 Hyperpigmented Skin/Oral Lesions	7 (10%)
HAART Status at Time of KS Diagnosis		
	Naïve to HAART	34 (48.6%)
	IRIS	18 (25.7%)
	On HAART > 12 months	16 (22.9%)
	Previously Defaulted off HAART	2 (2.9%)
Median Absolute CD4 Count at Time of KS Diagnosis (range)		368 (3–1039)
CD4 Suppression at KS Diagnosis (WHO Definitions by Age), n = 60		
	None-Mild Immune Suppression	27 (45%)
	Advanced Immune Suppression	16 (26.7%)
	Severe Immune Suppression	17 (28.3%)
Presence of Concurrent Opportunistic Illness		32 (45.7%)
	Tuberculosis	25 (35.7%)
	Severe Malnutrition	12 (17.1%)
Cytopenias at Time of KS Diagnosis (n = 67)		
	Median Platelet Count (range)	201 (6–672)
	Platelet Count < 50	14 (20.9%)
	Platelet Count < 100	19 (28.4%)
	Median Hemoglobin (range)	8.8 (1.8–13.6)
	Hemoglobin < 6	13 (19.4%)
	Hemoglobin < 8	25 (37.3%)
	Median Absolute Neutrophil Count (range)	1953 (300–5930)
	Absolute Neutrophil Count < 500	2 (2.9%)
	Absolute Neutrophil Count < 1000	12 (17.9%)
ACTG TIS Staging Classification		
	T1 (n = 68)	42 (61.8%)
	I1 (n = 60)	17 (28.3%)
	S1 (n = 67)	32 (47.8%)

Among the 18 patients who presented with bulging lymphadenopathy and none of the prototypical KS skin/oral lesions or woody edema, 13 (72%) diagnoses were established by biopsy. Of the five patients who were diagnosed clinically, all of them presented with massive, bulging, firm lymphadenopathy in the neck and inguinal regions plus/minus the axilla, with lymph node masses measuring > 5cm in diameter in each patient.

Baseline CD4 count samples were available for 60 of 70 patients and 17 (28.3%) had severe CD4 suppression. Moderate-severe cytopenias at the time of KS diagnosis was a common finding in this cohort; 67 of 70 patients had baseline full blood counts. At the time of KS diagnosis, 28.4% of children had moderate thrombocytopenia (platelet count < 100 x 10^9^/L) while 20.9% had severe thrombocytopenia (platelets < 50 x 10^9^/L). Moderate anemia (hemoglobin < 8 g/dL) was noted in 37.3% with severe anemia (hemoglobin < 6 g/dL) in 19.4%. Cytopenias promptly improved after initiation of chemotherapy, with 88% of patients with moderate-severe thrombocytopenia resolving within 2 weeks without transfusion.

Nearly half of this cohort (48.6%) was already on HAART at the time of KS diagnosis. Sixteen patients (22.9%) were on HAART for more than 1 year, an additional ten (14.2%) on HAART for 2–6 months, and eight (11.4%) were on HAART for less than 2 months at the time of the KS diagnosis. Focusing on patients with an apparent IRIS phenomenon, among the eight patients diagnosed within 2 months of HAART initiation, 4 were event-free survivors, 4 died (two from KS, three within one month of diagnosis), and none of them had visceral or disseminated disease. Of the ten patients diagnosed with KS on HAART for 2–6 months, 9 died (six from KS, two within one month of diagnosis) and only one survived; among the nine deaths, 3 patients had abdominal visceral disease and 2 presented with > 20 skin/oral lesions. All of the patients with pulmonary KS were naïve to HAART at time of KS diagnosis. Analysis of clinical variables associated with HAART status revealed a significant association with visceral disease and being naïve to HAART only. Follow-up immunological and virological data were not available to truly define the occurrence of IRIS. We did not observe any apparent improvement in KS from HAART alone, regardless of duration of time on HAART.

### Response to Chemotherapy

**Fig 3:**
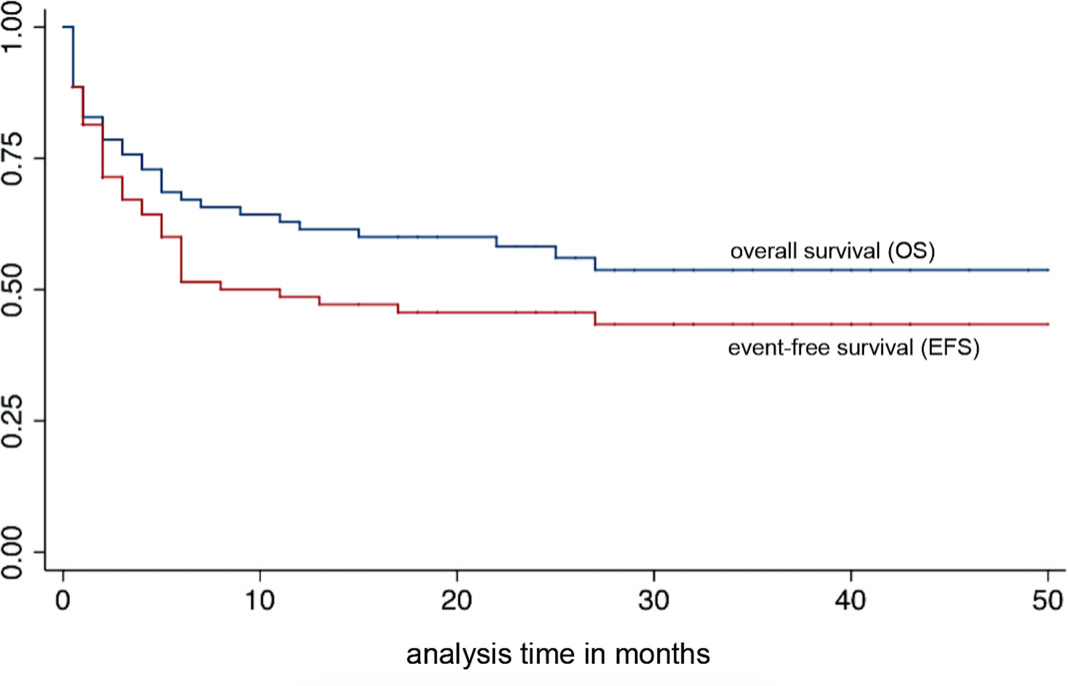
Kaplan-Meier curves demonstrating probability of overall (OS) and event-free survival (EFS) of 70 children and adolescents with KS.

With a median follow-up of 29 months (range 15–50), the 2-year OS was 58.2%. Long-term (median 31 months, range 15–50) CR was achieved in 33 children (47.1%), with an additional six patients (8.6%) alive with stable disease. Two-year EFS was 45.7% for the overall cohort (Fig 3). Relapse due to primary failure of the HAART regimen, with successful re-induction of CR after switching to 2^nd^ line HAART plus additional doses of chemotherapy, was not considered an event; this occurred in four patients with lymphadenopathic disease. Median time to event occurrence was 2 months (range 0.1–27 months). Five patients (7%) abandoned treatment; three were traced by telephone communication and found to have died, while two returned to care.

Fifty patients (71%) completed the full course of BV. The response to chemotherapy was often prompt, with resolution of KS lesions within 2–4 cycles of BV. Fourteen patients (20%) received less than the 4 cycles of induction BV; two abandoned therapy early, while 12 experienced early death. Of the 56 patients who completed induction BV, 32 (57%) achieved a CR or VGPR after induction. The response of woody edema to chemotherapy was notoriously slow; of the 14 patients with woody edema who completed induction BV, 12 (86%) were induction failures. Some patients with woody edema eventually experienced resolution of the edema, with the only remnant being subtle fibrotic knotty changes in the subcutaneous tissue, especially in the inner thigh. These patients were considered to be in CR if there was no residual edema and no asymmetry in the circumference of the extremity. Eight patients received salvage chemotherapy with ABV and three also received paclitaxel subsequently. Three of the patients receiving 2^nd^/3^rd^ line chemotherapy were long-term survivors, one after ABV alone, two after ABV plus paclitaxel.

BV was well tolerated. Based upon CTCAE criteria, 18.6% of patients experienced grade 4 neutropenia and 35.7% grade 3 neutropenia. There were no occurrences of grade 3 or 4 thrombocytopenia. Eight patients (11.4%) experienced grade 2 constipation and one patient experienced grade 2 peripheral neuropathy from vincristine. Pulmonary function testing and echocardiograms were not available to assess end-organ damage from bleomycin and doxorubicin respectively. All patients that died from respiratory failure did so in the setting of severe pleural effusions, which is not a characteristic of bleomycin toxicity. No patients developed clinical signs of cardiopulmonary insufficiency throughout the study period and strict guidelines were in place to avoid surpassing the total lifetime cumulative dosing limits of bleomycin and doxorubicin as outlined above.

Death occurred at a median time of 3 months after KS diagnosis (range 0.1–27 months). The majority of deaths (23 of 31, 74.2%) occurred less than 6 months from KS diagnosis. Median time to death for patients who died from progressive KS (n = 19) was 3 months (range 0.1–22, interquartile range 1–4 months), while median time to death for patients who died from other causes (multifactorial in patients that presented with KS and multiple other opportunistic illnesses [OI, n = 5], severe infection [n = 4], complications of severe malnutrition [n = 2], and unknown [n = 1]) was 3.5 months (range 0.1–27, interquartile range 1–6 months).

Ten patients died within 1–2 weeks of presentation; five had clinically refractory and progressive KS, however the other five (7.1%) presented with multiple acute severe OI and the cause of death is uncertain and therefore presumed treatment-related mortality (TRM). Six patients died in CR, three of them while on chemotherapy (one from presumed sepsis without neutropenia, one from bacterial meningitis without neutropenia, and one from complications of severe malnutrition). The latter two had poor control of their underlying HIV and it is difficult to definitively determine if their causes of death were treatment related versus secondary to uncontrolled HIV infection. Three patients died in CR long after completing chemotherapy (11, 15, and 27 months from KS diagnosis), all in the setting of poorly controlled HIV—one each from complications of severe malnutrition, severe pneumonia, and presumed sepsis.

### Risk Factors Associated with Death

Univariate logistic regression analysis was performed evaluating the risk of death and the risk of failure to achieve EFS in the cohort (Table 2). The following clinical variables demonstrated the strongest association with risk of death: visceral involvement (odds ratio (OR) 19, p < 0.01) and presenting with > 20 skin/oral lesions (OR 9.5, p = 0.01). Notable variables that were not associated with an increased risk of death included moderate-severe cytopenias, severe immune suppression, age, and T_1_ TIS tumor stage classification.

**Table 2:** Univariate Analysis of Variables Associated with Risk of Death and of Experiencing an Event.

Variable		Risk of Death			Risk of Experiencing an Event		
		Odds Ratio	95% CI	p value	Odds Ratio	95% CI	p value
Male Gender		1.42	0.55–3.65	0.47	1.12	0.44–2.9	0.81
Age ≥ 6 years		1.12	0.41–3.05	0.83	2.31	0.84–6.34	0.11
Age ≥ 12 years		0.45	0.14–1.46	0.18	1.70	0.55–5.27	0.36
Site of KS Presentation							
	Skin Involvement	2.61	0.94–1.07	0.06	2.62	0.97–7.07	0.05
	More than 20 Skin/Oral Lesions	9.50	1.08–83.86	0.01	15.00	0.82–273.98	0.07
	Lymph Node Involvement	0.83	0.27–2.48	0.73	0.28	0.08–0.99	0.03
	Oral Involvement	3.50	1.17–10.41	0.02	7.56	1.96–29.19	< 0.01
	Woody Edema	0.88	0.29–2.68	0.83	3.51	1.01–12.20	0.05
	Facial Edema	0.70	0.19–2.67	0.6	0.63	0.17–2.31	0.49
	Subcutaneous Nodules	0.32	0.11–0.97	0.04	0.28	0.10–0.81	0.02
	Inguinal Region Involvement	1.18	0.30–4.63	0.81	0.79	0.20–3.09	0.73
	Lymph Node Only Presentation	0.26	0.08–0.91	0.02	0.14	0.04–0.49	< 0.01
	Visceral Involvement	19.00	2.27–159.20	< 0.01	10.71	1.29–89.19	< 0.01
ACTG TIS Staging Classification							
	T1 vs T0	2.00	0.73–5.45	0.17	3.40	1.24–9.34	0.02
	I1 vs I0	1.78	0.56–5.61	0.33	1.57	0.50–4.91	0.43
	S1 vs S0	4.22	1.50–11.89	< 0.01	1.98	0.75–5.26	0.17
Induction Failure of BV x 4 Cycles		3.02	0.95–9.65	0.06	6.21	1.93–19.99	< 0.01
Moderate-Severe Cytopenias at time of KS Diagnosis							
	Hemoglobin < 8 g/dL	1.83	0.54–6.21	0.33	1.50	0.55–4.09	0.43
	Hemoglobin < 6 g/dL	2.55	0.92–7.04	0.07	1.49	0.43–5.13	0.53
	Platelet Count < 100 x 10^9/L	1.52	0.47–4.98	0.49	1.27	0.43–3.70	0.67
	Platelet Count < 50 x 10^9/L	1.85	0.63–5.42	0.26	0.83	0.25–2.69	0.75
	Absolute Neutrophil Count < 1,000 cells/mm3	1.32	0.38–4.59	0.66	0.76	0.22–2.63	0.66

The univariate analysis of variables associated with a failure to achieve EFS demonstrated very similar findings (Table 2). Important findings included the association of woody edema with failure to achieve EFS (OR 3.51, p = 0.05), and the favorable association of lymph node involvement (OR 0.28, p = 0.03). T_1_ staging did prove adversely associated with EFS (OR 3.4, p = 0.02), along with failure of induction phase BV (OR 6.21, p < 0.01). Using the log-rank test, there were no statistically significant associations between HAART status at the time of KS diagnosis and OS (p = 0.40) or EFS (0.50).

Multivariable analysis (Table 3) revealed that the only independent variables associated with a significant risk of death were visceral involvement and having more than 20 skin/oral lesions (OR 19.08, p = 0.007 and OR 9.57, p = 0.049 respectively). Failure to achieve EFS was independently predicted by visceral involvement, having more than 20 skin/oral lesions, and woody edema (OR 11.61, p = 0.015, OR 22.90, p = 0.05 and OR 7.80, p = 0.005 respectively). Oral involvement was not an independent risk factor.

**Table 3:** Multivariate Analysis of Variables Associated with Risk of Death and of Experiencing an Event.

Variable	Risk of Death			Risk of Experiencing an Event		
	Odds Ratio	95% CI	p value	Odds Ratio	95% CI	p value
More than 20 Skin/Oral Lesions	9.57	1.01–90.99	0.049	22.90	1.00–524.13	0.05
Visceral Involvement	19.08	2.22–163.90	0.007	11.61	1.60–83.95	0.015
Woody Edema	not significant			7.80	1.84–33.08	0.005
Subcutaneous Nodules	not significant			0.23	0.06–0.85	0.028

## Discussion

Analysis of detailed records from a retrospective pediatric KS cohort in Lilongwe, Malawi demonstrated that while lymphadenopathic KS was associated with improved long-term complete remission, visceral disease and disseminated skin/oral lesions were associated with a poor response to BV chemotherapy. Prior to a heightened awareness of lymphadenopathic KS in HIV-infected children, delays in diagnosis often resulted in patient deaths for lack of initiating proper KS-directed chemotherapy.[37] Instead, patients were usually misdiagnosed and mistakenly treated for tuberculosis and other infectious etiologies of lymphadenopathy. As demonstrated by the univariate risk analyses, children with lymphadenopathic KS demonstrate favorable outcomes when treated with BV; therefore greater identification of lymph node disease in this cohort may explain the superior 12-month OS in comparison to our published historical control (61% vs 43%).[10] This study highlights the virtually curative potential of a subset of HIV-infected children and adolescents with KS and suggests pathways towards better implementation of pediatric KS care in sub-Saharan Africa.

Very few studies on pediatric KS cohorts in sub-Saharan Africa exist, with most of them reporting only 1-year outcome data.[10–13] As a result, our knowledge of the long-term response to treatment is limited to and often based upon extrapolation from experiences in adult patients in the region. Benefiting from the integration of pediatric oncology and HIV services, our Pediatric HIV-Malignancy Program at the MW-COE has the advantage of long-term follow-up of HIV-infected patients that consistently return for refills of HAART. Of the 39 survivors in this cohort, 26 were in CR with greater than two years follow-up; the longest duration of follow-up was greater than four years.

Similar to other pediatric KS reports from sub-Saharan Africa, lymphadenopathy was the most common clinical presentation (74.3%) in our cohort as well. It is well established that the immunology of pediatric HIV infection is distinct from that in adults.[38] Early HIV infection and immune suppression of a naïve immune system may lead to unchecked primary HHV-8 lytic-phase infection, potentially explaining this phenomenon in children, which is seldom seen in adults who acquire HIV infection into a presumably competent immune system that has most likely been previously exposed to HHV-8 in regions with endemic prevalence.

Other unique features of pediatric KS demonstrated in our cohort include the low prevalence of severe CD4 suppression (28%), and the high rates of presentation with moderate-severe cytopenias. Low prevalence of severe CD4 suppression has also been observed in adult patients in Malawi.[39] Meanwhile, although anemia has been described in adult KS cohorts in Africa, the percentage of patients presenting with hgb < 8 g/dL (37%) in this pediatric cohort far exceeds that of adult descriptions.[40] Additionally, the high rate of presentation with severe thrombocytopenia (21%) in this cohort is a phenomenon never reported in adult KS patients. We theorize once again that lytic-phase HHV-8 infection may contribute to this phenomenon in HIV-infected children akin to the clinical patterns of other lytic-phase HHV-8-associated malignancies such as multicentric Castleman disease (MCD) and the KSHV inflammatory cytokine syndrome (KICS).[41–43] Bone marrow descriptions in patients with MCD reveal normocellular marrows with reactive plasmacytosis consistent with excess interleukin-6 (IL-6) or its HHV-8-encoded homolog, viral IL-6.[44] Although none of the patients in this cohort had a bone marrow evaluation, the near-uniform normalization of the cytopenias within 2 weeks of starting BV chemotherapy may lend credence to the theory of a cytokine-mediated pathophysiology. Ultimately, severe cytopenias were not associated with adverse outcomes.

Regarding KS staging, our data suggest that the adult KS T_1_ versus T_0_ staging classification is not significantly associated with higher mortality rates in children. Although presentation with visceral disease was a significant risk factor for death, other features of the T_1_ staging definitions such as woody edema were not. Conversely, although patients with widespread skin disease are classified as T_0_, those presenting with > 20 skin/oral lesions demonstrated high mortality rates in our cohort. Only 2 of the 7 patients with > 20 skin/oral lesions also had visceral disease, and the multivariable analysis revealed that both visceral disease and having > 20 skin/oral lesions are independent risk factors for EFS and OS. T_1_ staging though, did have a significant predictive value for the failure to achieve EFS in children. S_1_ staging demonstrated a higher risk for death though, and certainly the presence of another OI—especially severe malnutrition—renders these patients vulnerable to infectious, cardiovascular, and metabolic complications.

Selecting a threshold of 20 hyperpigmented skin/oral lesions was an arbitrary decision made retrospectively based upon evaluation of our historical experience in Lilongwe. While adult descriptions of KS often include much higher numbers of skin lesions (ie > 50), we found it a rare occurrence in our pediatric cohort.[34] Observing the patterns of clinical behavior in our patients based upon the descriptions of their original presentation, we decided upon a threshold of 20. These criteria will require prospective validation in larger clinical trials to become a universal definition of “disseminated” skin disease in children and adolescents. As such, we consider the ultimate decision on the threshold to define disseminated disease a dynamic issue that may be modified as the experience treating pediatric KS in Africa evolves.

Considering the practical limitations and challenges in low-income countries to prospectively identify all patients with visceral KS and other OI, it is important to monitor response to chemotherapy as well as empiric anti-microbial treatment. Although it is expected for woody edema to exhibit a slow response to BV chemotherapy, for all patients *without* woody edema, failure to respond to BV appears to be a poor prognostic sign and perhaps an indication for an alternative therapeutic approach. Patients with induction failure demonstrated an increased OR (6.21, p value < 0.01) for failure to achieve EFS in the univariate analysis, and represents an important variable to be evaluated in future, prospective clinical trials.

The lack of comprehensive pathology data represents an important limitation in the study. Although the clinical team is very experienced in the diagnosis of pediatric KS, uniform histological confirmation is always superior and is now the standard of care at KCH in Malawi. As mentioned above, making a definitive diagnosis of visceral KS is challenging with CXR and clinical evaluation as the only available resources. Additionally, 18 patients (26%) presented with lymphadenopathy and without the classical skin, oral, and edema KS lesions. While 13 of the 18 were confirmed with biopsy, the diagnosis was made clinically for five patients. The clinical diagnosis of lymphadenopathic KS in the absence of prototypical lesions presents a major challenge throughout the region. Although these five patients carrying clinical diagnoses of lymph node KS cannot be considered definitive, there were 13 confirmed lymph node biopsies and 34 other patients presenting with lymph node involvement plus prototypical KS skin/oral/edematous lesions, strengthening the pattern of observation of this distinct clinical presentation in eastern and central Africa, for which clinical descriptions (and images) actually date back to the 1960–70’s.[45, 46]

The five patients with a clinical diagnosis of lymph node KS presented with massive lymphadenopathy at the outset. Four presented with severe thrombocytopenia (range 13–24 x 10^9^/L) and without availability of platelets for transfusion, biopsies could not safely be performed considering the risk of bleeding. The fifth patient did not have severe thrombocytopenia, however he had already been on anti-tuberculosis treatment for more than 1 month and experienced worsening of all of the enlarged lymph nodes. Taking into account the severity of their clinical presentations, our historical observations of high mortality rates in lymphadenopathic KS patients experiencing delayed initiation of chemotherapy, their lack of improvement on anti-tuberculosis therapy (albeit of short duration), and the safety profile of the BV chemotherapy regimen, these patients were empirically treated for KS. We recognize that our threshold for the clinical diagnosis was low, however this stems from our historical experience of witnessing rapid clinical demise in especially those patients presenting with severe cytopenias. Three of the five patients remain in active follow-up in long-term CR. The other two both died; one from sepsis 6 weeks into treatment in a clinical CR, while the other died from severe pneumonia one day after starting chemotherapy.

While these five clinical diagnoses remain probable, we gave consideration to the following differential: generalized lymphadenopathy associated with HIV infection rarely presents as massively enlarged nodes, and patients generally are not critically ill. Although the duration of empiric anti-tuberculosis treatment was only 2 weeks or greater (and not sufficient to definitively determine failure of therapy), four of the patients clinically worsened despite it. The other most likely diagnoses on the differential would be a mature B-cell non-Hodgkin lymphoma or Hodgkin lymphoma, both of which cannot be adequately treated by merely bleomycin and vincristine alone. Therefore, with a retrospective lens and long-term follow-up, we feel that these five patients represent probable diagnoses of lymphadenopathic KS.

Limitations in pathology resources (i.e. exhaustive panels of immunohistochemical stains, flow cytometry, and HHV-8 virologic assays) also present a significant hurdle to establishing the co-existence of other HHV-8 malignancies such as primary effusion lymphoma, MCD, and KICS. This represents an important limitation in an HHV-8 endemic region with a high prevalence of pediatric HIV, where it would be logical for these other HHV-8 associated malignancies to occur. The retrospective aspect of the current study is another limitation. However, ultimately this manuscript presents a comprehensive and detailed account of the clinical characteristics, response to treatment, and long-term outcomes in pediatric KS and the data may serve to guide strategic planning for future clinical trials or the implementation of treatment paradigms.

## Conclusions

In conclusion, the combination of BV plus HAART was well tolerated, with minimal severe adverse events in a cohort of complex pediatric patients. The 2-year EFS of 45.7% and consistent long-term follow-up demonstrate that long-term CR can be achieved in pediatric KS. Lymphadenopathic KS is the most common clinical presentation in children in eastern Africa and is associated with good outcomes. Patients with woody edema have increased risk of failure to achieve EFS, but not mortality, ultimately exhibiting a more chronic disease course. However, patients with visceral disease and/or >20 widespread “disseminated” skin/oral lesions demonstrated significantly higher rates of death with BV; identifying these patients is critical to determining which patients will require alternative therapeutic strategies.
